# Preventive Orthodontia

**Published:** 1919-10

**Authors:** Ramond J. Wenker


### PREVENTIVE ORTHODONTIA

BY RAMOND J. WENKER, M.D., D.D.S.

It is indeed difficult to decide how early this field of practice may begin. Perhaps a good point to begin with would be with the prospective mother, in the form of an educational propaganda. I do not wish to discuss in detail what this propaganda should consist of, but in general it should include a consideration of the physical, mental and moral requirements for the mother to acquire and maintain her health and the health of her child. Every mother should be informed how to live to bring a healthy child into the world. She should know that no greater honor and privilege can be bestowed on her than that of giving birth to a healthy child. And to this end she should be taught sane, hygienic and rational methods of living, that she may substitute them for her superstitious and falacious ideas.

This phase of the subject overlaps that of the pediatrist's, but I can see no good reason why this propaganda should not be a combined movement including all parties interested and qualified to join in the undertaking.

One of the earliest active factors which influences growth is that of infection of the mother occurring prior to or during pregnancy. The most important of these is probably syphilis.

If a fetus becomes infected with syphilis it may die and be aborted. If perchance the syphilitic mother should develop a degree of immunity against the *treponema pallidum* the next pregnancy may result in the birth of a living child. Or if, by reason of anti-syphilitic treatment of the mother, the infection is reduced the future pregnancies offer more encouragement. The recognition and treatment of this infection is indicated. The physician in charge will be the one called upon, and his duty it will be to diagnose and treat not only the child, but the father and mother as well. The infection, however, may be so mild as to be overlooked.

Evidences of syphilis may frequently be recognized by the orthodontist in early childhood. The manifestations common in the new born are coryza, mucus patches, and skin eruptions. When these heal they leave scars, especially at the angles of the mouth and nose. Other manifestations later in life may be: affected sight, hearing, gummata in various parts of the mouth, Hutchison teeth, etc. Tardy eruption and extensive cavies are also conspicuous in this affection.

Congenital defects, namely, hare-lip and cleft-palate, are important affections interfering with normal jaw development and should receive early treatment. These children are frequently weak and delicate, and require the care of the skilled oral surgeon. Treatment of these cases may be regarded as preventive measures in the field of orthodontia.

The acute infectious diseases of childhood are important causative factors in the development of dentofacial malrelations. These affections are of interest to the orthodontist from the point of view that they favor or produce hyperplasia of the lymphoid tissue of the nasopharynx and also cause a general prevention of metabolism, and thus influence the growth of the child. Every general metabolic disturbance has a corresponding influence in jaw development, especially during the early stages of growth. The usual treatment is indicated in these affections.

Federspiel has suggested that pituitary disturbances may have a bearing on jaw development to a greater extent than we have heretofore recognized.

There are two types of pituitary disturbances which may occur in childhood: the one is hyper-pituitarism, which produces excessive growth, and the other is hypopituitarism, which has the opposite effect resembling cretinism. In disturbances of the thyroid gland there is but one type which is of interest to the orthodontist, and that is hypo-thyroidism, known as cretinism.

All bone dystrophies due to disturbances of the endocrine glands should receive organotherapy and other rational treatment according to indications.

It has been evident for many years that the nutrition of the child in the first months of life is of great importance in relation to future growth. It appears that a given handicap to normal metabolism is more harmful in proportion as it occurs earlier in life.

Dietary considerations are therefore the next in order, and although the physician in charge usually attends to this requirement, still the orthodontist should be familiar with this subject, that he may intelligently discuss it with the parents or physician in its relation to jaw development.

Williams, an authority on obstetrics, has submitted evidence of the infrequency of normal nursing of infants. He makes the statement that less than 25 per cent. of the mothers of New York State are able to nurse their children, and that that number is decreasing. Hellman contends that arrested development of the jaws is more prevalent among artificially fed infants than among the breast fed. He has estimated that 81 per cent. of arrested jaw development may be found in the artificial class. In the author's study of 643 children he has found 508 cases of mal development of the jaw in the artificial class, or a trifle over 79 per cent. That there is a large percentage of arrested development among this class is very evident to the observing orthodontist. This may be accounted for in the different proportion of the ingredients and digestible quality of cow's milk and other substitutes as compared with human milk.

Another interesting and valuable point in connection with artificial feeding is the greater susceptibility to infection among these children than those of the breast-fed class. This may be accounted for in the prevalence of defective metabolism as well as the absence of the immunity imparted by human milk. The adenoid type is therefore more prevalent also in this class.

There are two preventive measures which suggest themselves to the author which should be resorted to by the mother not able, or apparently unable, to nurse her children. The first choice is to encourage her organism to secrete milk by means of diet, exercise and massage—and the other is the old practice of employing a good wet-nurse. Both of these methods have proven very successful.

If, however, artificial feeding must be resorted to, there are two important points to determine. First, the digestive capacity; and second, the caloric requirement of the infant. The former should not be overtaxed in the effort to bring the quality and quantity of the food up to the caloric requirement. To this end the entire dependence on any single or any combination of proprietary foods is contra-indicated because of the large carbo-hydrate content and the deficiency of fat and protein which they usually contain.

In the author's estimation, rachitis is one of the most important dietary disturbances in the entire list. I believe it is a much more active causative factor than it is generally conceded to be. Heretofore we have only recognized it in its most pronounced form. As a matter of fact, there are a great variety of minor manifestations, and particularly that of arrested jaw development, which have in a large measure been overlooked. Lischer has called our attention to mal development of the mandible resulting from rickets. Holt and Fischer have also mentioned it as a factor, but in a rather general way. In the author's studies and clinical observation it appears that in every case of rachitis, with all of the gross classical symptoms there is usually a marked jaw deformity, equally as pronounced as the case illustrated by Lischer. There is also a large percentage of cases with minor general bone involvement which manifest comparatively mild forms of jaw deformities. Dewey has called our attention to the early shedding of deciduous teeth and tardy eruption of the permanent teeth in rachitic children. The absence of mechanical stimulus to the jaws supplied by erupted deciduous teeth from the time of shedding until the eruption of the permanent teeth must have a detrimental effect on their growth. Noyes has discussed this mechanical stimulus imparted to the growing jaws by the presence of the teeth. There is also manifested in rickets a tardy eruption of the *deciduous* teeth, which frequently causes much difficulty, such as inflammation, pain and suppuration. The artificially-fed class, however, does not supply all of the cases of rickets. Among the Italian and Ethiopian races a large number has been observed in the breast-fed class by Hess. In his experiments with these cases he has used cod liver oil with success as a corrective and preventive measure. For further information on this subject I would refer you to these authors.

Parents should be made to realize the full value of the services of the orthodontist from birth to early adult life to aid in the prevention of jaw deformities. His counsel in conjunction with the physician in charge should be of real value, provided, of course, that the orthodontist equips himself with the necessary knowledge to make his services valuable. He should be well informed on all questions pertaining to infant metabolism. He should be especially prepared to explain all phases of jaw development and tooth eruption and to take care of delayed and abnormal eruption. All dietary errors and pernicious habits should be detected early and steps taken for their correction. The child should be encouraged to use its teeth as soon as they erupt. To this end, it should be encouraged to chew crusts of bread and other hard material, not for the food value, but for the exercise it may get in the effort. I believe strongly in the value of mouth gymnastics, beginning as early as possible and continuing during the entire growing period. Much credit is due to Rogers for his valuable discussion of muscular exercise in corrective orthodontia. It is likewise very valuable in preventive orthodontia. The stimulating influence to the circulation and digestion, and therefore to normal metabolism, imparted by this method is very helpful. If, however, the supply of food material is deficient as to quality, no amount of exercise will serve as a substitute for good food. Converting them into play has proven quite successful, especially in the hands of a clever mother. Another method is to reward the child for carrying out the exercises. As the child progresses in development it is, of course, understood that its teeth should receive regular prophylactic treatment. They should be kept filled and hygienic. In fact, the entire mouth as well as the nose and throat should be kept in good health during the growing period.

A very important branch of preventive orthodontia, namely, the diagnosis and treatment of adenoids and troublesome tonsils, has been largely neglected by the orthodontist. If preventive measures are to be practiced in part, may I ask the question: why should not all preventive treatment be included? If the orthodontist is not prepared to do so, has he made ample preparation to practice his specialty? I contend that he should be prepared to practice all reasonable and legitimate phases of it.

Bogue, of New York, has called our attention to the frequency of arrested development of the jaws in early childhood. He maintains that the deciduous teeth should show a definite spacing between the ages of two and six. In other words, nature should anticipate and get ready for the eruption and alignment of the larger set of teeth which she is growing in the deeper structures. This spacing should become manifest as early as two or three years of age by the expansive growth of the jaws larger than is necessary to accommodate the deciduous teeth. When it is most pronounced it may be observed in all parts of the arch. The widest spacing usually appears in the anterior part of the arch. In less marked cases it is evident between the anterior teeth only. Unfortunately, however, this spacing is absent, or is evident only in slight degree, in an extremely large percentage of the children of the present period. Bogue states that from 94 to 95 per cent. of the children of the present period present evidences of arrested development of the jaws from two to six years of age. He has also called our attention to a normal minimum size of deciduous jaws. They should not measure less than 28 millimeters in width between the lingual surfaces of the molars on one side to the same location across the arch. All jaws above this width manifest a definite spacing, and all below show an absence of or deficient spacing. While we are disinclined to accept a definite measurement as indicative of the normal or abnormal, still a minimum measurement may serve the practical purpose of indicating, in the average case, the dividing line between the two classes.

The large percentage of arrested development of the deciduous jaws which Bogue has estimated, I presume, is calculated on this minimum basis. This percentage appears excessive; nevertheless, we all agree that there is a marked prevalence of arrested development in the present period.

Arrested development of the jaws is also to be observed in the adult. Abundant evidence of it is constantly before the mouth, nose and throat specialist. In an examination of the mouth, nose and throat of 2,800 recruits for the U. S. Army, the author has observed arrested development or deficient width of the jaws in fully 90 per cent. The cause of this condition was not ascertained, but the great prevalence of it was carefully noted during this examination.

What relation there may be between the large percentage of bottle-fed children, the large number of rachitic cases, and the large percentage of arrested jaw development is impossible to correctly state. The 75 percentage of Williams, the 81 percentage of Hellman, the 78 and 90 percentage of the author, and the 94 percentage of Bogue are at least suggestive of a relationship.

These figures are, however, subject to modification by making a study of a still larger number of cases and by making due allowance for numerous other conditions. Racial, climatic, social and economic conditions have their influence, and these have not received due consideration in a study sufficiently elaborate to make them final. We may therefore accept them as bearing only a tentative relationship.

The treatment of these cases should consist of both *preventive* and *corrective* treatment. The question is: "To what extent can these arrested and mal developments be prevented?" It is probable that much can be done by an educational propaganda among mothers, as previously mentioned. This propaganda should in the course of years effect a large reduction in the number of these deformities.

As soon as this arrested development can be diagnosed, namely, between the ages of two and six, if the health and stamina of the child will permit, expansion of the jaws may be undertaken. Unfortunately, a very large percentage of these children are unmanageable because of an unstable nervous system and harmful teaching. Much depends on the stamina of the child, and still more upon the skill of the orthodontist. If, however, a given child can not be managed the only alternative is to postpone the treatment.

The question of method may be briefly considered at this point. The Richardson plate, or some modification of it, seems the most feasible. The modification which appears practical to the author for this purpose is a removable basket, lingual arch, or the new Jackson appliance secured by means of bands cemented to the teeth, provided with lugs or tubes by means of which to automatically lock the appliance in place. This may be made so secure that the child can not remove it, but the operator can.

The next step is the predetermination of the permanent arch in order to serve as a guide in the expansion of the deciduous arch. This method, however, which I am about to describe, is not intended as a scientific correlation, but rather as a suggestion based on esthetic correlation. If expansion is undertaken before the eruption of the permanent anterior teeth, a radiograph of the upper central, lateral and cuspid on the side may be taken to determine their total width. Using this measurement as the radius, draw a circle with a pair of calipers to represent the curvature of the upper permanent arch from the distal surface of one cuspid to the distal of the other. Then enlarge the X-ray of the labial aspect of the central crown by photographic means until the labio-gingival curvature corresponds in size to the arch of the circle. This enlarged photograph of the central is then cut out on a line corresponding with the gingival curvature and approximal sides. Place this cut photograph over the circle and its approximal sides furnishes a continuation of the arch from the distal surface of each cuspid distally. This completed arch line will serve as an approximate guide for the size and shape of the future permanent arch of a given individual.

The length of time necessary to retain a deciduous arch after expanding will vary according to whether the patient is giving active co-operation in muscular exercises, according to age, the type of case, and according to whether the arches have been overexpanded to allow for a certain amount of reaction of the tissues in contracting after removal of the appliance.

An educational propaganda on preventive orthodontia, including preventive pediatrics, should be organized on a large scale and should be composed of interested and qualified members among the laity as well as the professions. This organization should become a part of the general social service movement for better babies.

No field of educational activity can be a greater benefit to humanity than this.

A child with a defective initial growth is handicapped throughout life.

That the enormous prevalence of mal development may be materially reduced there can be no question.

That this prevalence is largely due to artificial feeding appears evident.

The hazard to jaw development by artificial feeding is not generally recognized, neither by the laity nor the medical profession. Artificial feeding should be freely and persistently discouraged and the danger of it impressed on mothers.

The orthodontist should become a valuable consultant and co-worker with the physician in pediatrics. This should begin with early infancy in preference to the custom of waiting until a deformity is recognized.—*Dental Items of Interest.*

